# Supplementary material for: Pressure and ultrasound activate mechanosensitive TRAAK K+ channels through increased membrane tension
Source: bioRxiv. 2023 Jan 12:2023.01.11.523644. Preprint. [Version 1] doi: 10.1101/2023.01.11.523644 (PMC9882092; doi:10.1101/2023.01.11.523644)
Supplement: 1 — Supplemental Figure 1. TRAAK and MscS are equivalently activated by tension generated by positive and negative pressure. A. Normalized current-pressure relationship for positive (periwinkle) and negative (green) pressure for a TRAAK-containing patch. B. Tension-pressure relationship from data in A. C. Normalized current-tension relationship for channels activated by positive (periwinkle) and negative (green) pressure. D-F. Same as A-B, but for a MscS-containing patch. Supplemental Figure 2. Ultrasound and pressure generate membrane tension to activate MscS channels A. (left) Overlaid macroscopic currents and (right) corresponding fluorescent images from a MscS-containing patch in response to pressure (periwinkle) and ultrasound (green) stimulation. Traces and images are compared from the same patch activated to high (upper) and moderate (lower) I/Imax. Fits (red) and determined membrane radii are indicated on the images. Similar patch radii are observed when ultrasound or pressure stimuli generate similar channel activation. B. Normalized current-patch radius relationship calculated from a single patch in response ultrasound and pressure stimulation. C. Comparison of patch radii observed during ultrasound or pressure stimulation that resulted in I/Imax values near 0.5. Data from 5 different patches is shown (p = 0.95, paired t-test, not significant). [file NIHPP2023.01.11.523644v1-supplement-1.pdf]

bioRxiv preprint doi: https://doi.org/10.1101/2023.01.11.523644; this version posted January 12, 2023. The copyright holder for this preprint (which was not certified by peer review) is the author/funder, who has granted bioRxiv a license to display the preprint in perpetuity. It is made available under aCC-BY 4.0 International license.

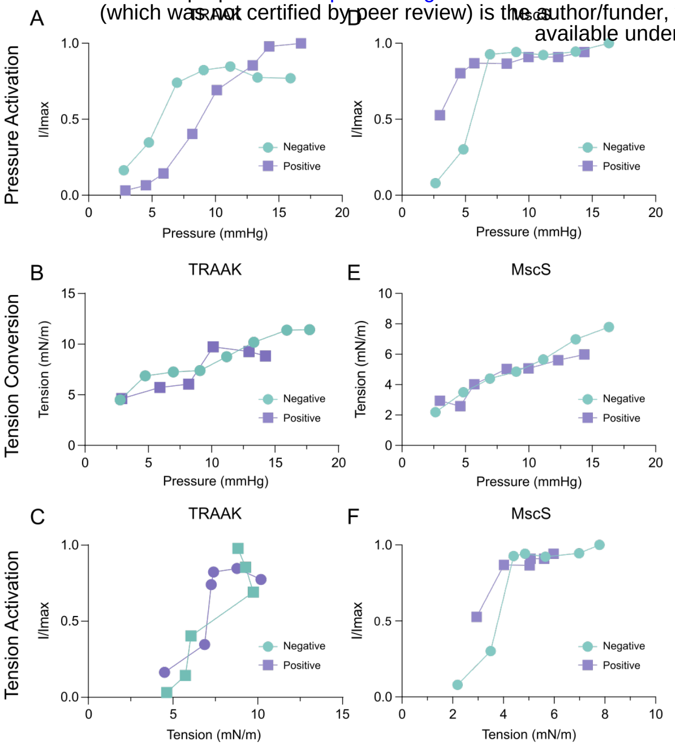

Supplemental Figure 1. TRAAK and MscS are equivalently activated by tension generated by positive and negative pressure.
A. Normalized current-pressure relationship for positive (periwinkle) and negative (green) pressure for a TRAAK-containing patch. B. Tension-pressure relationship from data in A. C. Normalized current-tension relationship for channels activated by positive (periwinkle) and negative (green) pressure. D-F. Same as A-B, but for a MscS-containing patch.

bioRxiv preprint doi: https://doi.org/10.1101/2023.01.11.523644; this version posted January 12, 2023. The copyright holder for this preprint (which was not certified by peer review) is the author/funder, who has granted bioRxiv a license to display the preprint in perpetuity. It is made available under aCC-BY 4.0 International license.

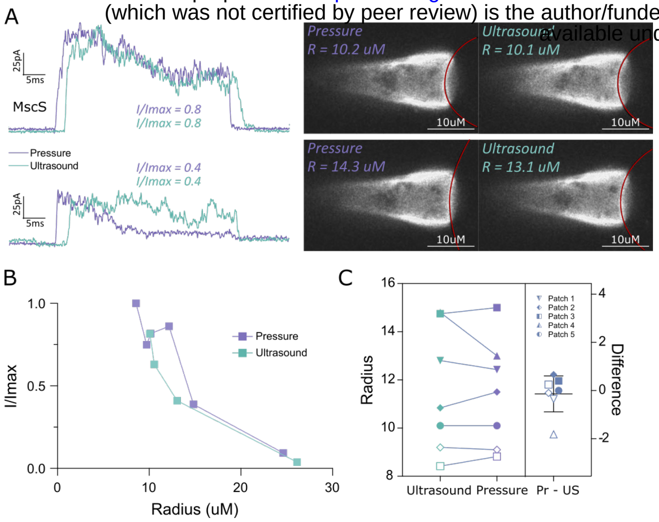

Supplemental Figure 2. Ultrasound and pressure generate membrane tension to activate MscS channels

A. (left) Overlaid macroscopic currents and (right) corresponding fluorescent images from a MscS-containing patch in response to pressure (periwinkle) and ultrasound (green) stimulation. Traces and images are compared from the same patch activated to high (upper) and moderate (lower) I/Imax. Fits (red) and determined membrane radii are indicated on the images. Similar patch radii are observed when ultrasound or pressure stimuli generate similar channel activation. B. Normalized current-patch radius relationship calculated from a single patch in response ultrasound and pressure stimulation. C. Comparison of patch radii observed during ultrasound or pressure stimulation that resulted in I/Imax values near 0.5. Data from 5 different patches is shown ($p = 0.95$, paired t-test, not significant).
